# Growth outcomes of small for gestational age preterm infants before and after implementation of an exclusive human milk-based diet

**DOI:** 10.1038/s41372-021-01082-x

**Published:** 2021-05-19

**Authors:** Lindsay Fleig, Joseph Hagan, Martin L. Lee, Steven A. Abrams, Keli M. Hawthorne, Amy B. Hair

**Affiliations:** 1 Department of Pediatrics, Division of Neonatology, University of Texas at Houston Health Science Center, Children’s Memorial Hermann Hospital, Houston, TX USA; 2 Department of Pediatrics, Section of Neonatology, Baylor College of Medicine, Texas Children’s Hospital, Houston, TX USA; 3 Prolacta Bioscience, Duarte, CA USA; 4 Department of Biostatistics, UCLA Fielding School of Public Health, Los Angeles, CA USA; 5 Department of Pediatrics, Dell Medical School, University of Texas at Austin, Austin, TX USA

**Keywords:** Paediatrics, Outcomes research

## Abstract

**Objective:**

Small for gestational age (SGA) preterm infants (PT) are at greatest risk for growth failure. Our objective was to assess the impact of an exclusive human milk diet (HUM) on growth velocities and neonatal morbidities from birth to discharge in a SGA population.

**Study design:**

Multicenter, retrospective cohort study, subgroup analysis of SGA PT comparing a cow’s milk diet (CMD) with HUM diet.

**Results:**

At birth 420 PT were classified as SGA (197 CMD group, 223 HUM group). Demographics and anthropometric measurements were similar. HUM group PT showed improvement in length *Z* score at discharge (*p* = 0.024) and reduction in necrotizing enterocolitis (NEC) (*p* = 0.004).

**Conclusion:**

SGA PT fed a HUM diet had significantly decreased incidence of NEC, surgical NEC, and late-onset sepsis. Due to concerns about growth in a HUM diet, it is reassuring SGA infants fed the HUM diet had similar growth to CMD diet with trends toward improvement.

## Introduction

Previously, our group published a multicenter retrospective cohort study comparing infants who received a cow’s milk diet (CMD) of mother’s own milk (MOM) fortified with cow’s milk-based fortifier (CMF) and/or preterm formula to infants who received an exclusive human milk diet (HUM) of MOM and/or donor human milk (DHM) fortified with human milk-based fortifier (HMBF) [1, 2]. This pre-post cohort study demonstrated improved outcomes in necrotizing enterocolitis (NEC), late-onset sepsis, and bronchopulmonary dysplasia (BPD) after implementation of the HUM diet. Infants born small for gestational age (SGA), defined as <10th percentile for weight, are at higher risk for postnatal growth failure at discharge [3–6]. Our previous study included both SGA and appropriate for gestational age (AGA) infants in their cohorts.

The American Academy of Pediatrics, Committee on Nutrition updated their policy statement in December 2017 on breastfeeding and DHM use specific for high-risk preterm infant populations [7]. DHM use is now widely used in the US for preterm infants weighing less than 1500 g birth weight (BW) when MOM is not available or is contraindicated. MOM is always preferred because of the known biological components affected by pasteurization used in DHM. There are significant short- and long-term beneficial effects of feeding preterm infants a HUM diet compared to a mixed CMD diet, including decreased late-onset sepsis, reduction in BPD, mortality, and decreased risk of developing NEC supported by multiple published studies comprising of randomized controlled trials [8–11].

However, there still remain concerns about the growth of infants receiving a HUM diet, as human milk must be fortified for the most high-risk and premature infants to achieve adequate nutrient intake [12–14]. Hair et al. illustrated that the use of a HUM diet was safe and did not negatively impact growth of infants at a single study site in infants <1250 g BW [15]. Human milk should be fortified with protein, minerals, and vitamins to ensure optimal nutrient infant for infants weighing <1500 g BW. It is established that both MOM and DHM have large variability in nutritional content in terms of calories per ounce, protein, carbohydrate, and fat content. Therefore, in order to avoid postnatal growth failure, a diet with human milk, either MOM or DHM, must be supplemented with fortification, either CMF or HMBF.

The primary aim of this study is to assess the impact of the HUM diet on anthropometric growth velocities from birth to discharge in the SGA infant population. The secondary outcomes assessed focus on the impact of the HUM diet on neonatal morbidities, specifically NEC and mortality, in this SGA infant population.

## Methods

In a previously published multicenter, retrospective cohort study, infants who received a CMD diet of MOM fortified with CMF and/or preterm formula were compared to growth of infants who received a newly introduced HUM diet feeding protocol consisting of MOM and/or DHM with HMBF [1, 2]. Groups compared were infants fed a CMD diet to infants fed a HUM diet. All infants <1250 g BW and SGA were included. SGA was defined as <10th percentile for weight in grams at birth [3]. Excluded infants had major congenital anomalies, died in the first 12 h of life or were transferred in from an outside hospital after 7 days of life. The Institutional Review Board at each study site approved this retrospective study.

### Study outcomes

Study data were collected for approximately 2 years prior to and after introduction of a HUM diet. Primary data were extracted from the electronic medical record and included infant diet, demographics, growth parameters, and outcomes. Each study site had detailed documentation of feeding protocol and time frame for data collection [1]. Participating centers were Baylor College of Medicine (BCM) (Houston, TX), Good Samaritan San Jose Hospital (GSH) (San Jose, CA), Northwestern Prentice Women’s Hospital (Chicago, IL), and Winnie Palmer Hospital (Orlando, FL). BCM was the largest trial contributor with 62% infants; however, all sites were level 4 NICUs in both academic and private institutions. There were differences among the sites with respect to specific feeding protocols documented in detail. Full feedings were considered 140–160 ml/kg/day [1]. At BCM in the HUM diet group, enteral feeds were started with trophic feeds of 20 ml/kg/day for 3 days, and advanced by 20 ml/kg/day as tolerated to goal of 140–160 ml/kg/day. HMBF was added (Prolacta Biosciences, Industry, CA) at 60 ml/kg/day volume for an additional 4 kcal/oz, at 100 ml/kg/day at for an additional 6 kcal/oz, and if weight gain was <15 g/kg/day, an additional 8–10 kcal/oz was provided. At BCM in the CMD diet group, fortification did not start until feeds were at 100 ml/kg/day. At the GSH, the HUM group feeds were fortified at 100 ml/kg/day with 4 kcal/oz, and then at 150 ml/kg/day an additional 6 kcal/oz was provided, and if growth failure, addition of 8–10 kcal/oz was provided. The CMD diet group at GSH followed the same protocol as BCM. At Northwestern and Winne Palmer Hospital, the HUM diet group was fortified with an additional 4 kcal/oz once feeds reached 100–120 ml/kg/day, and advanced to 6 kcal/oz if weight gain was <15 g/kg/day, and further advanced to 8 kcal/oz. In the CMD diet group at Northwestern followed the same protocol as BCM. At Winne Palmer Hospital, the CMD diet group was fortified approximately at 120–150 ml/kg/day. All infants, regardless of whether SGA or AGA, received the same feeding protocols at each institution, and no specific feeding protocol was followed for SGA infants.

The original primary study outcomes have been previously published for the entire cohort consisting of both SGA and AGA infants [1]. In this subgroup analysis of the original data, we investigated the SGA cohort alone specific for growth and neonatal outcomes. Growth velocities from birth to discharge were calculated and compared between CMD diet and HUM diet groups. Weight velocity was calculated in in grams per kilogram per day (g/kg/day) using the exponential method [16]. Length velocity was calculated in centimeters per day (cm/day). Head circumference (HC) velocity was calculated in centimeters per day (cm/day). *Z* scores were calculated for the growth velocities using either Fenton growth curves if infants were discharged home before 50 weeks’ postmenstrual age (PMA) or World Health Organization growth curves for infants discharged home after 50 weeks’ PMA [3, 17]. Growth data were adjusted for length of stay, antenatal steroids, and by study site.

### Outcomes defined

PMA at discharge was defined as the number of weeks and days added to gestational age at birth. As described by the Fenton growth curve, SGA was defined as BW in grams <10th percentile and AGA was defined as BW in grams between the 10th and 90th percentiles [3]. Common neonatal outcomes measured included NEC defined as Bell’s stage IIA or greater with presence of pneumatosis intestinalis on abdominal radiograph reviewed by a pediatric radiologist [18]. Surgical NEC was defined as NEC requiring surgical intervention within the acute phase of illness [1]. All NEC cases were reviewed individually at time of data collection to delineate between spontaneous intestinal perforations versus NEC. BPD was defined as mild BPD in infants <32 weeks’ gestation with need for supplemental oxygen for greater than or equal to 28 days, moderate BPD was defined as requiring supplemental oxygen for greater than or equal to 28 days with less than 30% FiO_2_ at 36 weeks’ PMA, and severe BPD was defined as requiring supplemental oxygen for greater than or equal to 28 days with greater than or equal to 30% FiO_2_ or positive pressure at 36 weeks’ PMA [19]. Late-onset sepsis was defined as a positive blood culture obtained after 72 h of life. Patent ductus arteriosus (PDA) was measured by echocardiogram read by a board-certified pediatric cardiologist. Severe intraventricular hemorrhage was diagnosed by head ultrasound read by a board-certified pediatric radiologist using the Papile grading method [20].

### Statistical analysis

The distributions of quantitative variables were summarized using the median and interquartile range with diet group comparisons made using the Wilcoxon rank-sum test. Categorical variables were compared between the study groups using Fisher’s exact test. A 5% significance level was used for all comparisons.

## Results

Of the original 1587 infants among the four study centers, there were 420 infants who were classified as less than the 10th percentile, defined with Fenton growth curves, included in this analysis [1, 3]. There were 197 infants in the CMD group, pre-initiation of the HUM feeding protocol, and 223 infants in the HUM group, post initiation of the HUM feeding protocol. Infant demographics and anthropometric measurements were similar (Table 1).

**Table 1:** Small for gestational age (SGA) infants’ characteristics (*n* = 420).

	CMD (*n* = 197)	HUM (*n* = 223)	*p* value
Male^a^	104 (53)	116 (52)	0.922
Race^a^			
Black	72 (37)	76 (34)	0.610
Hispanic	46 (23)	45 (20)	0.477
White	63 (32)	86 (39)	0.184
Other	16 (8)	16 (7)	0.717
Gestational age (weeks)^b^	28.0 (26.0, 30.2)	28.0 (26.0, 30.0)	0.516
Birth weight (g)^b^	705.0 (566.0, 923.0)	760.0 (580.0, 965.0)	0.215
Birth weight *Z* score^b^	–1.5 (–1.8, –1.2)	–1.4 (–1.8, –1.1)	0.778
Birth length (cm)^b^	32.0 (30.0, 35.0)	33.0 (30.5, 36.0)	0.193
Birth length *Z* score^b^	–1.6 (–2.3, –1.0)	–1.6 (–2.2, –1.0)	0.594
Birth head circumference (cm)^b^	23.5 (21.5, 26.0)	24.0 (21.5, 26.0)	0.497
Birth head circumference *Z* score^b^	–1.4 (–2.1, –1.0)	–1.5 (–2.0, –0.9)	0.712
Multiple gestation^a^	40 (20)	55 (25)	0.297
SGA at birth^a^	197 (100)	223 (100)	1.000
Antenatal steroids^a^	162 (82)	187 (84)	0.697

^a^Frequency (%), Fisher’s exact test *p* value.

^b^Median (interquartile range), Wilcoxon rank-sum test *p* value.

The primary outcomes for growth measurements between the CMD and HUM groups are shown in Table 2. Length discharge *Z* score was greater on the average in the HUM group (*p* = 0.024). Although not significant, all other parameters (velocity, discharge *Z* score, and change in *Z* score from birth to discharge) for weight, length, and HC were numerically superior in the HUM group. Diagnosis of SGA at discharge was not significantly different between the groups (*p* = 0.14). Of the original 197 infants in the CMD group, 168 infants (85%) remained SGA at discharge and of the original 223 infants in the HUM group, 201 (90%) remained SGA at discharge. Length of stay was not significantly different between the groups (*p* = 0.91).

**Table 2:** Small for gestational age (SGA) infants’ primary outcomes for growth parameters (*n* = 420).

	CMD (*n* = 197)	HUM (*n* = 223)	*p* value
Discharge weight (g)^a^	2385.0 (1920.0, 3255.0)	2390.0 (1980.0, 3045.0)	0.598
Discharge weight *Z* score^a^	–2.4 (–3.2, –1.6)	–2.3 (–3.1, –1.8)	0.829
Weight Δ *Z* score^a^	–0.9 (–1.5, –0.1)	–0.8 (–1.3, –0.4)	0.915
Weight velocity (g/d)^a^	19.6 (15.8, 23.2)	19.7 (16.5, 22.6)	0.843
Discharge length (cm)^a^	44.5 (42.0, 47.5)	45.0 (42.5, 47.5)	0.263
Discharge length *Z* score^a^	–3.3 (–4.4, –2.2)	–3.0 (–3.9, –2.0)	0.024
Length Δ *Z* score^a^	–1.4 (–2.7, –0.4)	–1.2 (–2.1, –0.4)	0.174
Length velocity (cm/week)^a^	0.9 (0.7, 1.0)	0.9 (0.8, 1.1)	0.210
Discharge head circumference (cm)^a^	32.5 (30.6, 35.3)	33.0 (31.0, 35.0)	0.828
Discharge head circumference *Z* score^a^	–1.6 (–2.4, –0.8)	–1.5 (–2.6, –0.7)	0.563
Head circumference Δ *Z* score^a^	0.0 (–0.9, 0.8)	–0.1 (–1.0, 0.4)	0.286
Head circumference velocity (cm/week)^a^	0.7 (0.6, 0.8)	0.7 (0.6, 0.8)	0.179
SGA at birth^a^	197 (100)	223 (100)	1.000
SGA at discharge^a^	168 (85)	201 (90)	0.137
Postmenstrual age at discharge (weeks)^a^	39.6 (37.2, 43.8)	39.9 (37.4, 43.4)	0.901
Length of stay (days)^a^	77.0 (48.0, 121.0)	77.0 (48.0, 121.0)	0.913

^a^Median (interquartile range), Wilcoxon rank-sum test *p* value.

The neonatal morbidities for the CMD and HUM groups are presented in Table 3. Among the SGA infants in this study, late-onset sepsis (*p* = 0.017), NEC (*p* = 0.004), and surgical NEC (0.045) were significantly lower in the HUM group. In our SGA population, the number needed to treat is 11.1 infants to prevent one case of NEC and 20 infants to prevent one case of surgical NEC. Death was similar among the groups with 36 deaths (18.2%) in the CMD group compared to 37 deaths (16.6%) in the HUM group (*p* = 0.70). In addition, PDA (*p* = 0.078), retinopathy of prematurity at any stage (*p* = 0.18), and BPD (*p* = 0.27) rates were lower in the HUM group, but not significantly so. The HUM group required a median of 2 fewer mechanical ventilation days (*p* = 0.12).

**Table 3:** Small for gestational age (SGA) infants’ secondary outcomes for neonatal morbidities (*n* = 420).

	CMD (*n* = 197)	HUM (*n* = 223)	*p* value
NEC^a^	33 (17)	17 (8)	0.004
Surgical NEC^a^	18 (9)	9 (4)	0.045
PDA^a^	105 (53)	99 (44)	0.078
ROP, any stage^a^	13 (7)	8 (4)	0.182
Severe IVH^a^	15 (8)	15 (7)	0.850
Death^a^	36 (18.2)	37 (16.6)	0.699
BPD^a^	81 (41)	79 (35)	0.268
Mechanical vent days^b^	9.0 (2.0, 43.0)	7.0 (0.0, 41.0)	0.121
Late-onset sepsis^a^	53 (27)	38 (17)	0.017

^a^Frequency (%), Fisher’s exact test *p* value.

^b^Median (interquartile range), Wilcoxon rank-sum test *p* value.

## Discussion

Use of a HUM diet in SGA infants improved length discharge *Z* score, as evidenced previously in other studies [15, 21]. In addition, use of a HUM diet in SGA infants showed decreased late-onset sepsis, medical and surgical NEC cases, thus providing further validation for the HUM diet to reduce NEC [8, 22].

This study is unique in that it is the first analysis in the SGA infant population comparing growth and neonatal morbidities at multiple institutions after implementation of a feeding protocol using the HUM diet. This study provides insight into differences among SGA infants and may provide evidence to target this high-risk population for a HUM diet. Multiple institutions and the relatively large number of infants enrolled in each group allows for increased generalizability of our results.

### Growth outcomes in SGA infants

Further investigation into how the HUM diet impacts our most fragile and small infants, the SGA population, requires sincere equipoise. Controversy in evidence exists with respect to the HUM diet and growth failure. In a smaller retrospective study in a single level III NICU, infants <1000 g BW fed the HUM diet had improved feeding intolerance but persistent growth failure despite adjustment for SGA [23]. SGA infants in this study were not evaluated separately from all extremely low birth weight (ELBW) infants. In addition, in Eibensteiner et al., 192 ELBW infants were evaluated at multiple centers and found no improvement in growth or neonatal morbidities, again without distinction of the SGA population [24]. SGA infants are at a higher risk for growth failure, long-term metabolic outcomes, higher risk for NEC and feeding intolerance, and therefore, should be evaluated separately. In contrast to these findings with specific focus on the SGA population by Hair et al., premature SGA infants fed the HUM diet exhibited greater catch-up growth without negative metabolic outcomes as compared to AGA infants [25]. In addition, premature SGA infants fed a HUM diet show lower insulin levels, no difference in adiposity in the groups, and improvement in body composition at 2-year follow-up [25]. Sullivan et al. showed that there was no difference in growth between infants fed HUM and infants fed a mixed CMD diet [8]. The ultimate goal in premature neonatal nutrition is to optimize enteral nutrition without increasing the risk of neonatal morbidities and mortality, such as life-threatening NEC. In-hospital growth is associated with long-term neurodevelopmental outcomes and improving growth may reduce overall morbidity associated with prematurity [26]. A systematic review illustrated improvement of neurodevelopmental outcomes with visual and cognitive benefits into adolescence with the increasing dose received of MOM in preterm infants [27].

The use of a HUM diet in infants <1250 g BW (both SGA and AGA) is associated with a lower rate of NEC and decreased parenteral nutrition days [1, 8, 9, 11]. However, there remains a paucity of evidence in the literature regarding a direct comparison of MOM fortified with CMF to the HUM in the most high-risk and fragile SGA premature infant population.

Postnatal growth failure is a common complication of prematurity and SGA infants are at even higher risk. There are concerns that infants classified as SGA at birth will have a high incidence of postnatal growth failure [26]. In a cohort of 1776 SGA infants, 97% of them remained SGA at 36 weeks’ corrected age [5]. Similarly in our cohort, growth failure persisted at discharge in both groups, with a slightly improved relative reduction in SGA in the CMD group compared to HUM group, although the difference did not achieve statistical significance. Despite this smaller reduction in SGA at discharge, both groups showed similar growth. The SGA infants fed the HUM diet showed possible trends toward improvement, with only the length discharge *Z* score being statistically significant. These clinically improved growth outcomes, although not statistically significant, in addition to decreased mortality support the use of DHM in the high-risk infant when MOM is not available and further supports the use of early and rapid advancement of fortification with HMBF for growth [8, 25].

In a study by Dusick et al., infants born with in utero growth failure less than 1000 g BW had a higher incidence of growth failure postnatally when compared to AGA [4]. Similarly, in a large retrospective study of 24,371 infants by Clark et al., growth restriction at discharge is inversely related to younger gestational age and weight at birth [6]. However, another study reports similar growth velocities when comparing SGA to AGA infants, perhaps because of factors other than fortification and attainment of full feedings [15]. These concerns for the SGA infant population make them of unique concern for further specialized investigation.

### Neonatal morbidities in SGA infants

The reduction of NEC with use of a HUM diet evidenced in larger infants remains consistent in our cohort of SGA infants [1, 8, 9, 11]. In this cohort there were 53% fewer cases of NEC in the HUM group. It is suspected that the effect of cow’s milk in the premature infant diet is dose dependent with fewer days to full feedings and fewer ventilator days as the percentage of CMD decreased. Therefore, limiting exposure to CMF in the SGA population should be considered [22]. Moreover, significantly decreased incidence of late-onset sepsis was evident in the HUM group, consistent with previous reports [1].

### Limitations

This study is limited by it being a secondary analysis of previously collected data gathered retrospectively and potentially unidentified time-dependent care changes. In addition, a retrospective study may have misclassification of NEC diagnosis, although the diagnosis was made by two separate physicians in the original study’s Bell’s Staging Criteria [18]. Despite these limitations, a significant difference in length discharge *Z* score was evident, and other parameters favored the HUM group.

## Conclusion

In this secondary analysis of a retrospective cohort study of SGA infants after the initiation of a HUM feeding protocol, those who received a HUM diet had significantly decreased incidence of NEC, surgical NEC, and late-onset sepsis. Although SGA infants fed a HUM diet had similar growth to CMD infants for most outcomes, length was improved in the HUM group. Due to concerns about growth in infants who receive a HUM diet, it is reassuring that SGA infants had similar growth to CMD with some possible trends toward improvement. Further investigation into the specialized needs of SGA infants to overcome postnatal growth failure is needed.
